# Specific aromatic foldamers potently inhibit spontaneous and seeded Aβ42 and Aβ43 fibril assembly

**DOI:** 10.1042/BJ20131609

**Published:** 2014-10-23

**Authors:** Katelyn M. Seither, Heather A. McMahon, Nikita Singh, Hejia Wang, Mimi Cushman-Nick, Geronda L. Montalvo, William F. DeGrado, James Shorter

**Affiliations:** *Department of Biochemistry and Biophysics, Perelman School of Medicine at the University of Pennsylvania, 805b Stellar-Chance Laboratories, 422 Curie Boulevard, Philadelphia, PA 19104, U.S.A.; †Neuroscience Graduate Group, Perelman School of Medicine at the University of Pennsylvania, 805b Stellar-Chance Laboratories, 422 Curie Boulevard, Philadelphia, PA 19104, U.S.A.; ‡Department of Pharmaceutical Chemistry, University of California, San Francisco, CVRI-MC Box 3122, San Francisco, CA 94158-9001 U.S.A.; §Biochemistry and Molecular Biophysics Graduate Group, Perelman School of Medicine at the University of Pennsylvania, 805b Stellar-Chance Laboratories, 422 Curie Boulevard, Philadelphia, PA 19104, U.S.A.

**Keywords:** Alzheimer’s disease, amyloid, Aβ42 (amyloid-β 42), Aβ43 (amyloid-β 43), foldamer, protein misfolding, AD, Alzheimer’s disease, Aβ, amyloid-β, Benz, 3-amino benzoic acid, DCM, dichloromethane, DIEA, di-isopropylethylamine, DMEM, Dulbecco’s modified Eagle’s medium, DMF, dimethylformamide, EGCG, (−)-epigallocatechin-3-gallate, HFIP, 1,1,1,3,3,3-hexafluoro-2-propanol, LDH, lactate dehydrogenase, MeOH, methanol, NM, N-terminal and middle domains of Sup35, Sal, salicylamide, TEV, tobbaco etch virus, ThT, thioflavin-T

## Abstract

Amyloid fibrils are self-propagating entities that spread pathology in several devastating disorders including Alzheimer's disease (AD). In AD, amyloid-β (Aβ) peptides form extracellular plaques that contribute to cognitive decline. One potential therapeutic strategy is to develop inhibitors that prevent Aβ misfolding into proteotoxic conformers. Here, we design specific aromatic foldamers, synthetic polymers with an aromatic salicylamide (Sal) or 3-amino benzoic acid (Benz) backbone, short length (four repetitive units), basic arginine (Arg), lysine (Lys) or citrulline (Cit) side chains, and various N- and C-terminal groups that prevent spontaneous and seeded Aβ fibrillization. Ac-Sal-(Lys-Sal)_3_-CONH_2_ and Sal-(Lys-Sal)_3_-CONH_2_ selectively inhibited Aβ42 fibrillization, but were ineffective against Aβ43, an overlooked species that is highly neurotoxic and frequently deposited in AD brains. By contrast, (Arg-Benz)_4_-CONH_2_ and (Arg-Sal)_3_-(Cit-Sal)-CONH_2_ prevented spontaneous and seeded Aβ42 and Aβ43 fibrillization. Importantly, (Arg-Sal)_3_-(Cit-Sal)-CONH_2_ inhibited formation of toxic Aβ42 and Aβ43 oligomers and proteotoxicity. None of these foldamers inhibited Sup35 prionogenesis, but Sal-(Lys-Sal)_3_-CONH_2_ delayed aggregation of fused in sarcoma (FUS), an RNA-binding protein with a prion-like domain connected with amyotrophic lateral sclerosis and frontotemporal dementia. We establish that inhibitors of Aβ42 fibrillization do not necessarily inhibit Aβ43 fibrillization. Moreover, (Arg-Sal)_3_-(Cit-Sal)-CONH_2_ inhibits formation of toxic Aβ conformers and seeding activity, properties that could have therapeutic utility.

## Abstract 2

We report specific aromatic foldamers that inhibit fibrillization by amyloid-β (Aβ) peptides connected with Alzheimer's disease. One foldamer inhibits formation of toxic Aβ-species as well as the self-templating activity of Aβ fibrils, properties that could have therapeutic utility.

## INTRODUCTION

Protein misfolding can be fatal [1,2]. Proteins misfold from soluble species into highly stable, cross-β amyloid fibrils in Alzheimer's disease (AD) and several other neurodegenerative diseases [1,2]. One strategy to combat these disorders is to develop small molecules that inhibit amyloidogenesis and prevent toxic protein misfolding [3–6]. Although daunting challenges face potential small molecule inhibitors of amyloidogenesis [7], they are beginning to reach the clinic. Indeed, tafamidis, a small molecule inhibitor of transthyretin amyloidogenesis treats familial amyloid polyneuropathy, a rare but deadly disease [8,9].

Here, we focus on amyloid-β (Aβ) peptides, Aβ42 and Aβ43, which form amyloid fibrils and accumulate in extracellular plaques that are a hallmark of AD [10–16]. AD is a progressive neurodegenerative disease and the most common cause of dementia worldwide [12]. Aging is a significant risk factor for AD and there are no effective therapies [11]. In Aβ biogenesis, the full-length transmembrane amyloid precursor protein (APP) undergoes sequential cleavage by β- and γ-secretase, resulting in peptides that are 38–43 amino acids in length [10,12]. Aβ42 and Aβ40 are most commonly associated with AD pathology [10–12]. Aβ40 is a more benign, perhaps even neuroprotective species [17,18], which slowly assembles into amyloid fibrils. By contrast, Aβ42 oligomerizes and fibrillizes more rapidly due to two additional C-terminal residues that introduce additional steric zipper hexapeptides that drive assembly [19–21].

Although Aβ peptides longer than Aβ42 are found in AD, they are not a major species and their pathogenic role has been ignored. Recently, this view has changed. Aβ43 is a potent contributor to neurotoxicity in AD [13–15]. Aβ43 contains an additional threonine residue at the C-terminal end and fibrillizes more rapidly than Aβ42 [13]. Aβ43 is more abundant in insoluble fractions than Aβ40 in AD and its presence in senile plaques is directly correlated with cognitive decline [13–16]. Specific inhibitors of Aβ43 misfolding have not been identified and it is unclear whether inhibitors of Aβ42 misfolding will also inhibit Aβ43 misfolding.

Aβ monomers form amyloid via nucleated conformational conversion [22]. First, a subpopulation of Aβ monomers forms molten oligomers, which gradually rearrange into amyloidogenic oligomers that nucleate cross-β fibrils [22,23]. Rearrangement is rate limiting and causes the lag phase of spontaneous fibrillization [22]. During lag phase, Aβ forms diverse oligomeric species, which can be highly toxic [21,24–27]. Upon nucleation, fibrils rapidly grow via their self-templating ends, which convert Aβ conformers into the cross-β conformation [20,28]. When coupled to fibril fragmentation, this ‘seeding’ activity enables Aβ fibrils to become self-propagating agents that transmit pathology and disease [1,29–31]. Aβ fibrils also provide catalytic surfaces for ‘secondary’ nucleation events distinct from fibril elongation [32–34]. Here, lateral Aβ fibril surfaces convert Aβ monomers into toxic oligomers [32–34]. Thus, formation of toxic oligomers and fibrils is intimately linked [32–34]. These secondary nucleation events also help explain Aβ assembly kinetics [32–34]. Aβ forms different cross-β fibril structures termed ‘strains’, which can differ in toxicity and cause distinct brain pathology [35–38]. Aβ fibrils are usually less toxic than pre-amyloid oligomers [21,39]. However, Aβ fibrils also display toxicity [6,35,36,39]. A key challenge is to manipulate Aβ assembly in a manner that depopulates toxic conformers [7]. Agents that inhibit seeded assembly hold promise for preventing the spread of Aβ pathology in AD.

Numerous potential inhibitors of Aβ misfolding have been explored, including small molecules, peptides, molecular chaperones, protein disaggregases and antibodies [3,6,39–45]. In the present study, we explore a different strategy by pursuing foldamers; non-biological discrete chain molecules that lack a canonical peptide backbone but can fold into specific structures [46]. Foldamers have been utilized as antimicrobial agents and molecular scaffolds [47–50]. Peptides containing non-natural amino acids, similar to foldamers, have been useful for understanding the misfolding of various amyloidogenic peptides [42,51–53]. Foldamers have several advantageous properties that could make them a valuable class of amyloid inhibitors. Due to their semi-rigid backbone, foldamers can assume an organized conformation at low entropic cost with relatively few monomeric units [50,54]. Compared with α peptides, foldamers have greater thermodynamic stability and resist proteases. Furthermore, foldamers of varying lengths with diverse side chains and 3D shapes can be synthesized. These features enable foldamer design for interaction with diverse biological targets [47–50,55]. In the present study, we explore aromatic foldamers as antagonists of Aβ42 and Aβ43 amyloidogenesis.

## MATERIALS AND METHODS

### Generation of soluble and fibrillar Aβ42 and Aβ43

To produce monomeric Aβ, synthetic lyophilized Aβ42 or Aβ43 (W.M. Keck Facility, Yale University) was dissolved in 1,1,1,3,3,3-hexafluoro-2-propanol (HFIP, Sigma) at 2 mg/ml. HFIP was removed by drying in a speed vacuum for 30 min. The resulting peptide film was dissolved in DMSO to 1 mM. Aβ42 or Aβ43 fibrils for seeding experiments were prepared by diluting monomerized Aβ42 or Aβ43 in KHMD (150 mM KCl, 40 mM Hepes–KOH pH 7.4, 20 mM MgCl_2_ and 1 mM DTT) to 10 μM. This solution was incubated at 37°C for 3–5 days with agitation (700 r.p.m.) in an Eppendorf Thermomixer. For seeding experiments, preformed fibrils were briefly sonicated or vortex-mixed prior to use. We also prepared Aβ42 or Aβ43 using a protocol that avoids DMSO. Thus, Aβ42 or Aβ43 was dissolved in HFIP followed by evaporation of the solvent to dryness [56]. Dry peptide films were dissolved in a minimal volume of 60 mM NaOH followed by dilution with deionized water and sonication for 1 min using a bath sonicator. Peptides were diluted to 0.2 mM by adding an equal volume of 20 mM sodium phosphate buffer (PB, Sigma), pH 8 plus 0.2 mM EDTA (PBE). Samples were centrifuged at 16000 ***g*** for 3 min and subjected to Superdex 75 gel filtration in PBE to remove residual solvent.

### Foldamers

Foldamers (Lys-Sal)_4_-CONH_2_, (Arg-Benz)_4_-CONH_2_, (Lys-Sal)_4_-COMe, (Lys-Sal)_4_-COOH, (Lys-Sal)_4_-COβAla, Ac-(Lys-Sal)_3_-CONH_2_, Sal-(Lys-Sal)_3_-CONH_2_ and Ac-Sal-(Lys-Sal)_3_-CONH_2_ (where Sal is salicylamide and Benz is 3-amino benzoic acid) were from PolyMedix and were dissolved in TBS (50 mM Tris/HCl pH 7.4, 150 mM NaCl) to obtain concentrated stock solutions. Foldamers (Cit-Sal)_4_-CONH_2_, (Arg-Sal)_2_-(Cit-Sal)-(Arg-Sal)-CONH_2_, (Arg-Sal)_3_-(Cit-Sal)-CONH_2_, (Cit-Sal)_2_-(Arg-Sal)-(Cit-Sal)-CONH_2_, (Cit-Sal)-(Arg-Sal)-(Cit-Sal)_2_-CONH_2_ and (Arg-Sal-Cit-Sal)_2_-CONH_2_ were also from PolyMedix. These foldamers were dissolved in 1:1 TBS/DMSO to obtain concentrated stocks. Subsequent dilutions were made from these stocks to appropriate concentrations in KHMD or PBE.

Foldamers (Lys-Sal)_2_-CONH_2_, Ac-(Lys-Sal)_2_-CONH_2_, Sal-(Lys-Sal)_2_-CONH_2_, (Lys-Sal)_3_-CONH_2_ and Ac-(Lys-Sal)_3_-CONH_2_ were synthesized at room temperature on a 100 μmol scale using rink amide resin (GemScript Corporation, 0.6 mmol/g substitution) for support of alternating α- (Bachem) and aromatic amino acids. Resin was swelled in 100% dimethylformamide (DMF, Fisher Scientific) for 1 h, followed by a 30 min deprotection using 5% piperazine (Sigma–Aldrich) in DMF. The first residue was coupled to the resin using 3 equiv. of amino acid, 2.8 equiv. of 2-(6-chloro-1H-benzotriazole-1-yl)-1,1,3,3-tetramethylaminium hexafluorophosphate (HCTU, GL Biosciences) activator and 7.5 equiv. of di-isopropylethylamine (DIEA, CHEM-IMPEX International), shaking for 1 h at room temperature. The resin was washed three times each with DMF, dichloromethane (DCM, Fisher Scientific) and DMF. This step was followed by deprotection (as above). Coupling and deprotection steps were cycled for the remaining residues in each respective peptide sequence. After deprotection of the final residue the product was rinsed [three times with DMF, three times with DCM, three times with DMF and three times with methanol (MeOH)] and dried with MeOH. This product was split in half. The first half was re-swelled in DMF and acetylated by incubating the resin in 5% acetic anhydride in 2.5% DIEA and 92.5% DMF for 10 min. This acetylated portion was rinsed and dried (as above). Next, both halves (one with a N-terminal acetyl and a second with a N-terminal free amide) were cleaved from the resin using a cocktail of 2:2:2:94 H_2_O/TIS (tri-isopropyl silane)/anisole/TFA (trifluoroacetic acid; Sigma–Aldrich) for 2 h at room temperature. The peptide solution was filtered from the resin and precipitated using 1:1 cold ethyl ether:hexane. The precipitate was dried by lyophilization. The mass and purity of each product was verified by MALDI–TOF MS (Brucker microflex LRF) and analytical HPLC (C18 column). Dried crude foldamer was purified by preparative reverse-phase HPLC, dried by lyophilization and mass and purity was verified as above. All samples were prepared by directly dissolving lyophilized foldamer into TBS buffer to 2 mM.

### Spontaneous and seeded Aβ42, Aβ43 and N-terminal and middle domain of Sup35 (NM) fibrillization

For spontaneous fibrillization, soluble Aβ42 or Aβ43 (1 mM) in DMSO was diluted to 5 μM in KHMD containing 25 μM thioflavin-T (ThT) plus or minus foldamer (0–20 μM). For seeded fibrillization, preformed Aβ42 or Aβ43 fibrils (10 μM monomer) were added at a final concentration of 0.1 μM (monomer). Alternatively, Aβ42 or Aβ43 were prepared using just HFIP and were assembled at 5 μM in PBE containing 25 μM ThT plus or minus foldamer (20 μM). NM was purified as described [57]. NM (5 μM) was assembled in KHMD containing 25 μM ThT plus or minus foldamer (20 μM). For seeded fibrillization, preformed NM fibrils (5 μM monomer) were added at a final concentration of 0.1 μM (monomer). Reactions were conducted in 96-well plates and incubated at 25°C in a TECAN Safire II plate reader (Tecan USA) for up to 8 h with agitation. ThT fluorescence was measured at the indicated times. The excitation wavelength was 450 nm (5 nm bandwidth) and the emission wavelength was 482 nm (10 nm bandwidth). ThT fluorescence values reported are arbitrary and are normalized to the final assembly time point of the Aβ alone condition.

### FUS aggregation

GST–TEV–FUS was purified as described [58]. Aggregation was initiated by addition of tobbaco etch virus (TEV) protease to GST–TEV–FUS (5 μM) plus or minus foldamer (20 μM) in assembly buffer (50 mM Tris/HCl pH 8, 0.2 M trehalose and 20 mM glutathione). Aggregation was for 0–90 min at 25°C without agitation in a 96-well plate and was assessed by turbidity (absorbance at 395 nm) using a Tecan Infinite M1000 plate reader [58]. No aggregation occurred unless TEV protease was added to separate GST from FUS [58]. SDS/PAGE and Coomassie staining revealed that foldamers did not inhibit cleavage of GST–TEV–FUS by TEV.

### Electron microscopy

Reactions were adhered on to 300-mesh-formvar carbon-coated EM grids overnight before being negatively stained with 2% uranyl acetate for 2 min and rinsed with milli-Q distilled water. Micrographs were acquired using a JEOL 1010 TEM (Jeol USA).

### Tracking A11-reactive Aβ42 or Aβ43 conformers

The oligomer-specific A11 antibody was used to detect toxic Aβ42 or Aβ43 oligomers by ELISA as described [21]. Foldamers did not cross-react with A11.

### Toxicity assays

SH-SY5Y human neuroblastoma cells were maintained in Dulbecco's modified Eagle's medium (DMEM) plus 10 mM Hepes, 10% FBS, 4 mM glutamine, penicillin (200 units/ml) and streptomycin (200 μg/ml) in 5% CO_2_ at 37°C. Cells were differentiated in serum-free DMEM with N2 supplement and 10 μM all-*trans*-retinoic acid before use. Cells were plated at (10000 cells/well) in 96-well plates and grown overnight. Medium was removed and Aβ conformers or controls were added and cells were incubated for 24 h at 37°C. Toxicity was assessed using an MTT kit (Tox-1; Sigma) or via lactate dehydrogenase (LDH) release using the CytoTox-ONE™ kit (Promega). Toxicity values were normalized to the buffer control without Aβ.

## RESULTS AND DISCUSSION

### Rationale and foldamer design

**Figure 1:**
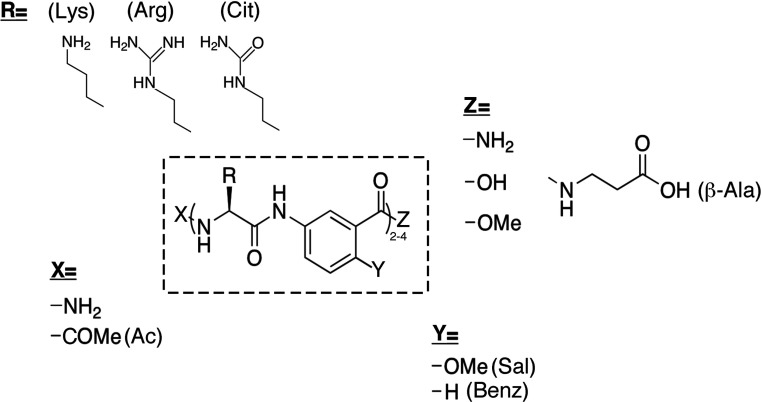
Overview of aromatic foldamer structure The core foldamer structure is shown in the dashed box, which can be decorated with different moieties at X-, R-, Y- and Z-positions indicated on the periphery. Foldamers possess an aromatic Sal or Benz backbone (Y=OMe or H), Arg, Lys or Cit side chains (R=Arg, Lys or Cit), short length (two to four repetitive units) and various N- (X=NH_2_ or Ac) and C- (Z=NH_2_, OH, OMe or β-Ala) terminal groups.

**Figure 2:**
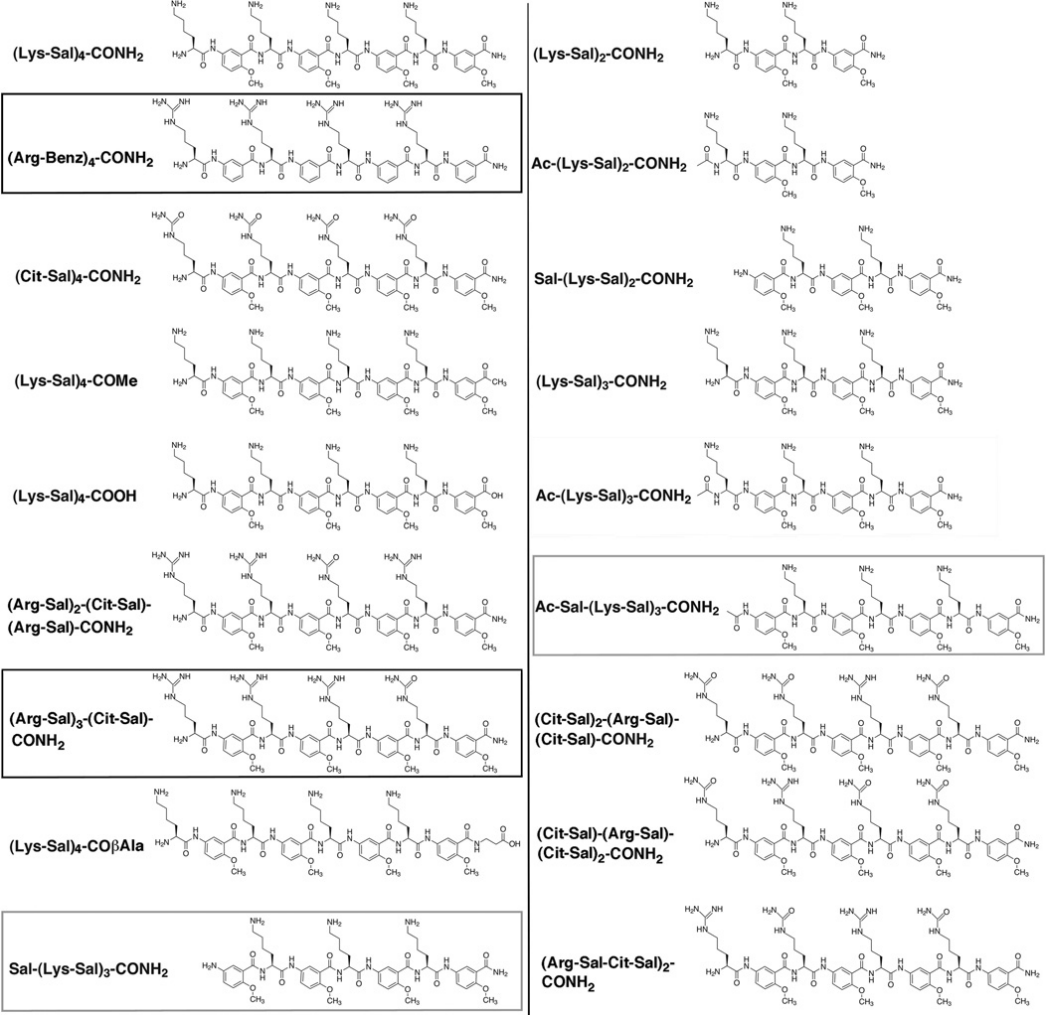
Nomenclature and structure of aromatic foldamers Three-letter amino acid nomenclature is used to indicate the side chain (Lys, Arg or Cit) and the Sal or Benz backbone is indicated. N- (Ac) and C- (NH_2_, OH, OMe or β-Ala) terminal groups are also indicated. Foldamers that inhibit spontaneous Aβ42 and Aβ43 fibrillization, (Arg-Benz)_4_-CONH_2_ and (Arg-Sal)_3_-(Cit-Sal)-CONH_2_, are boxed in black. Foldamers that inhibit spontaneous Aβ42 fibrillization but not spontaneous Aβ43 fibrillization, Sal-(Lys-Sal)_3_-CONH_2_ and Ac-Sal-(Lys-Sal)_3_-CONH_2_, are boxed in grey.

As potential inhibitors of Aβ42 and Aβ43 amyloidogenesis, we explored aromatic foldamers (Figures 1 and 2). Some of these foldamers were originally synthesized as inhibitors of heparin and are rich in aromatic and positively charged groups [55]. They possess an aromatic salicylamide (Sal) or 3-amino benzoic acid (Benz) backbone (Figure 1; Y=OMe or H), lysine (Lys), arginine (Arg) or citrulline (Cit) side chains (Figure 1; R=Lys, Arg or Cit), short length (two to four repetitive units) (Figure 1) and various N- (Figure 1; X=NH_2_ or COMe [Ac]) and C-(Figure 1; Z=NH_2_, OH, OMe or β-Ala) terminal groups. We selected this design for four reasons. First, the aromatic backbone is similar to ones employed by Nowick et al. [42,51–53] in protein aggregation inhibitors. Secondly, interactions between aromatic residues within short amyloidogenic peptides mediate molecular recognition during fibrillization [59]. Moreover, polyphenols such as (−)-epigallocatechin-3-gallate (EGCG) inhibit amyloidogenesis and prevent cytotoxicity [57,59–61]. Thus, the aromatic foldamer spine might antagonize aromatic interactions critical for fibrillization. Thirdly, the aromatic foldamers investigated are approximately the same length (two to four repetitive units) as steric zipper hexapeptides that form amyloid [19]. Finally, basic side chains, particularly arginine exert hydrotropic effects and prevent protein aggregation [62].

### Foldamer inhibition screen

**Figure 3:**
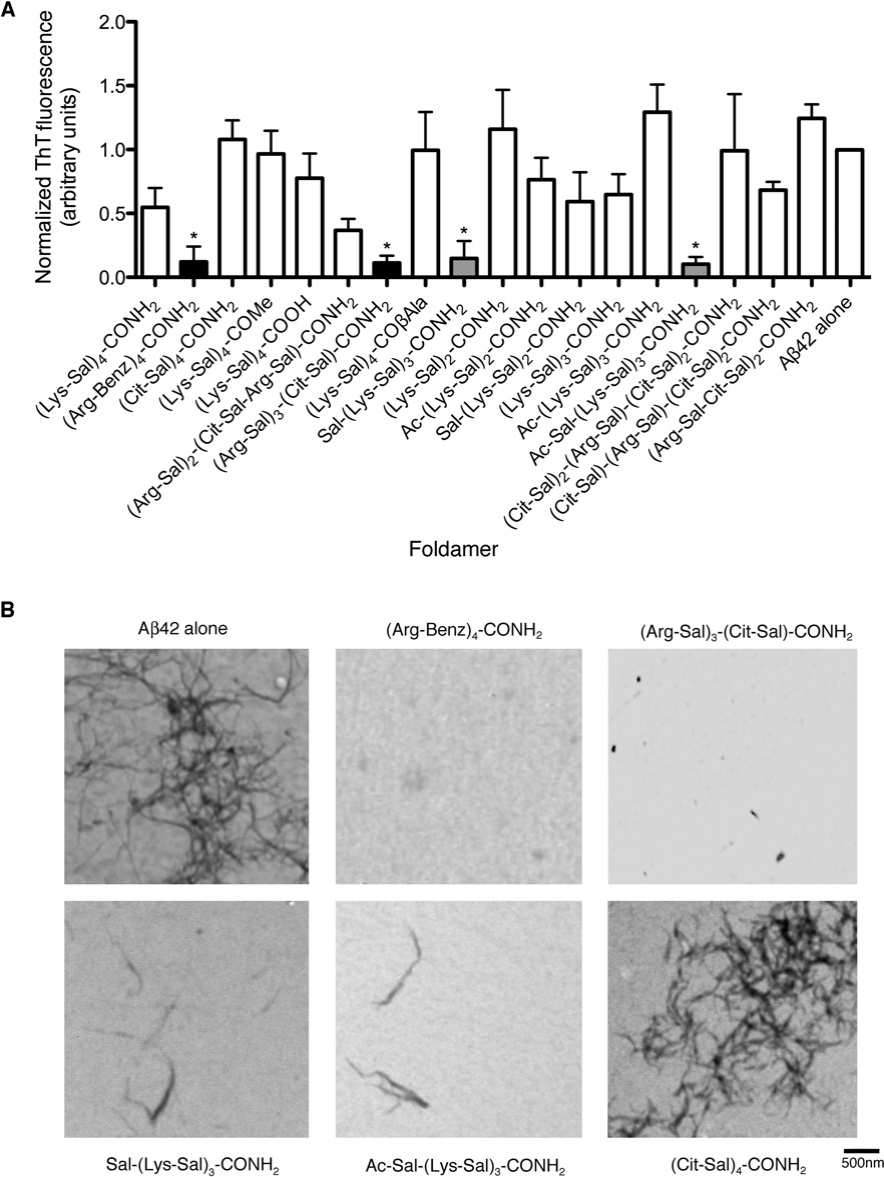
(Arg-Benz)_4_-CONH_2_, (Arg-Sal)_3_-(Cit-Sal)-CONH_2_, Sal-(Lys-Sal)_3_-CONH_2_ and Ac-Sal-(Lys-Sal)_3_-CONH_2_ inhibit spontaneous Aβ42 fibrillization (**A**) Aβ42 (5 μM) was incubated with agitation for 8 h at 25°C plus or minus the indicated foldamer (10 μM). Aβ42 fibrillization was assessed by ThT fluorescence. Values represent means±S.E.M. (*n*=3–6). A one-way ANOVA with the post-hoc Dunnett's multiple comparisons test was used to compare Aβ42 alone to each Aβ42 plus foldamer condition (* denotes *P*< 0.05). Foldamers that selectively inhibit Aβ42 fibrillization are indicated by grey bars and foldamers that inhibit Aβ42 and Aβ43 fibrillization are indicated by black bars. (**B**) Aβ42 (5 μM) was incubated with agitation for 4 h at 25°C in the absence or presence of the indicated foldamer (10 μM). Aβ42 fibrillization was assessed by EM. Scale bar, 500 nm.

**Figure 4:**
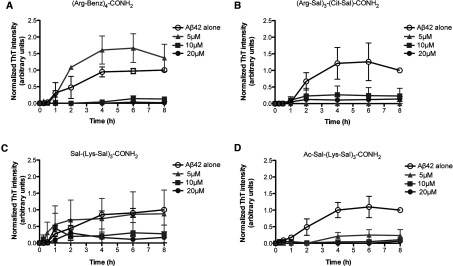
Effect of inhibitory foldamers on spontaneous Aβ42 fibrillization kinetics (**A**–**D**) Aβ42 (5 μM) was incubated with agitation for 0–8 h at 25°C in the absence (open circles) or presence of 5 μM (filled triangles), 10 μM (filled squares) or 20 μM (filled circles) (Arg-Benz)_4_-CONH_2_ (**A**), (Arg-Sal)_3_-(Cit-Sal)-CONH_2_ (**B**), Sal-(Lys-Sal)_3_-CONH_2_ (**C**) or Ac-Sal-(Lys-Sal)_3_-CONH_2_ (**D**). Aβ42 fibrillization was assessed by ThT fluorescence. Values represent means±S.E.M. (*n*=3).

We tested 18 aromatic foldamers (Figure 2) for inhibition of spontaneous (i.e. in the absence of preformed fibrils) Aβ42 fibrillization. The majority of foldamers did not significantly inhibit Aβ42 fibrillization (Figure 3A). However, (Arg-Benz)_4_-CONH_2_, (Arg-Sal)_3_-(Cit-Sal)-CONH_2_, Sal-(Lys-Sal)_3_-CONH_2_ and Ac-Sal-(Lys-Sal)_3_-CONH_2_ were strong inhibitors (Figure 2 boxed in black or grey; Figures 3A and 3B; Figures 4A–4D). (Arg-Sal)_3_-(Cit-Sal)-CONH_2_ was the most potent with an IC_50_ of ~1.6 μM.

Several important foldamer properties emerge for inhibition of Aβ42 fibrillization. First, a foldamer must have a backbone with at least four aromatic units to antagonize Aβ42 fibrillization. Thus, (Lys-Sal)_2_-CONH_2_, Ac-(Lys-Sal)_2_-CONH_2_, Sal-(Lys-Sal)_2_-CONH_2_, (Lys-Sal)_3_-CONH_2_ and Ac-(Lys-Sal)_3_-CONH_2_ failed to inhibit assembly (Figures 2 and 3A). Secondly, foldamers with more than three lysine or citrulline side chains were ineffective, encompassing: (Lys-Sal)_4_-CONH_2_, (Cit-Sal)_4_-CONH_2_, (Lys-Sal)_4_-COMe, (Lys-Sal)_4_-COOH and (Lys-Sal)_4_-COβAla (Figures 2 and 3A). By contrast, Sal-(Lys-Sal)_3_-CONH_2_ and Ac-Sal-(Lys-Sal)_3_-CONH_2_, which possess three lysine side chains and four aromatic backbone units, were potent inhibitors (Figures 2 and 3A). Thirdly, foldamers with three or more consecutive Arg side chains were effective inhibitors. Thus, (Arg-Benz)_4_-CONH_2_ and (Arg-Sal)_3_-(Cit-Sal)-CONH_2_ were potent inhibitors, whereas (Arg-Sal)_2_-(Cit-Sal)-(Arg-Sal)-CONH_2_, (Cit-Sal)_2_-(Arg-Sal)-(Cit-Sal)-CONH_2_, (Cit-Sal)-(Arg-Sal)-(Cit-Sal)_2_-CONH_2_ and (Arg-Sal-Cit-Sal)_2_-CONH_2_ were ineffective (Figures 2 and 3A).

Select small molecules that inhibit Aβ42 fibrillization also disassemble Aβ42 fibrils [4,57,60]. However, even when present in 4-fold molar excess, (Arg-Benz)_4_-CONH_2_, (Arg-Sal)_3_-(Cit-Sal)-CONH_2_, Sal-(Lys-Sal)_3_-CONH_2_ and Ac-Sal-(Lys-Sal)_3_-CONH_2_ did not disassemble Aβ42 fibrils after 24 h (results not shown). Thus, these foldamers do not reverse Aβ42 fibrillization.

### Foldamers that inhibit Aβ42 fibrillization do not inhibit NM fibrillization

**Figure 5:**
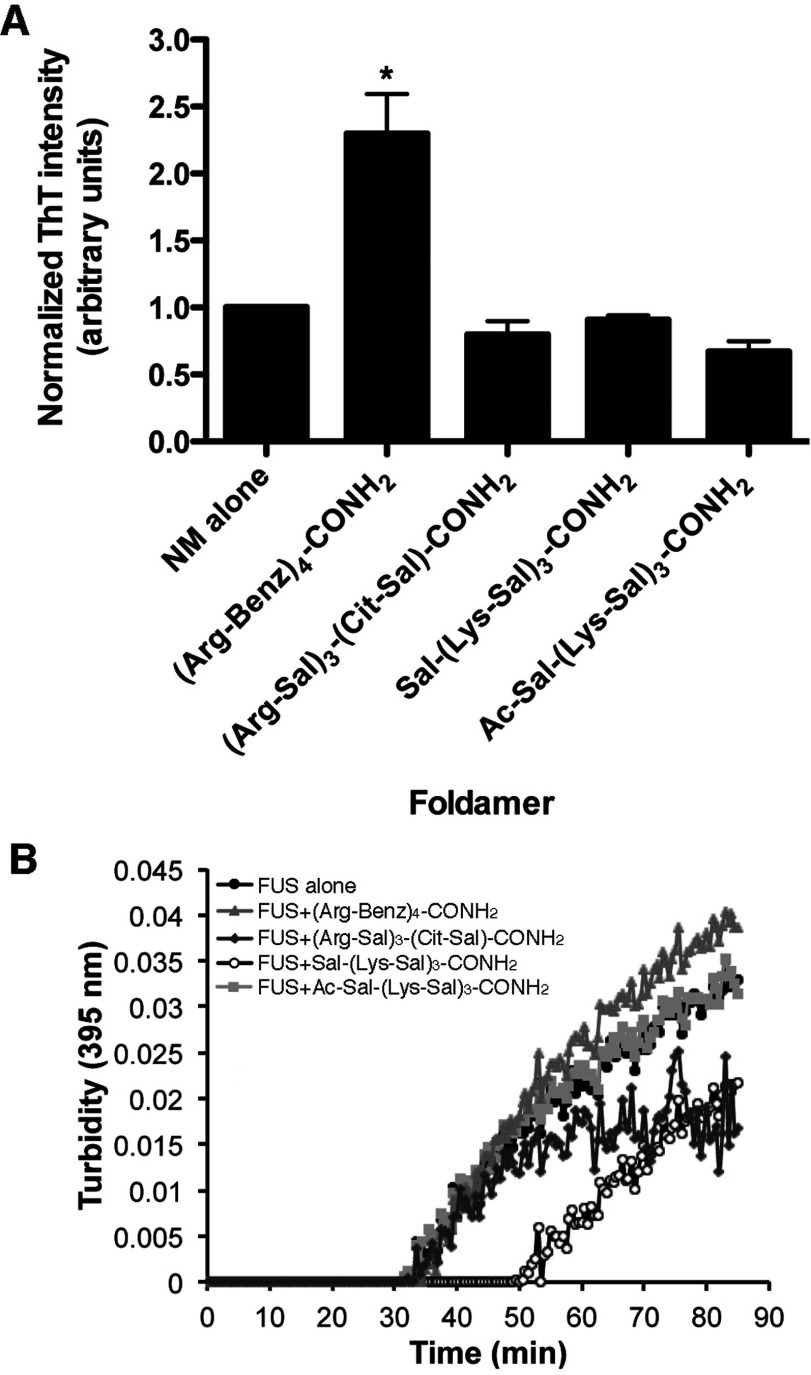
Sal-(Lys-Sal)_3_-CONH_2_ has no effect on NM fibrillization but delays FUS aggregation (**A**) NM (5 μM) was incubated with agitation for 4 h at 25°C plus or minus the indicated foldamer (20 μM). NM fibrillization was assessed by ThT fluorescence. Values represent means±S.E.M. (*n*=3). A one-way ANOVA with the *post-hoc* Dunnett's multiple comparisons test was used to compare NM alone to each NM plus foldamer condition (* denotes *P*< 0.05). (**B**) GST-FUS (5 μM) was incubated in the presence of the indicated foldamer (20 μM) plus TEV protease at 25°C for 0–90 min. Turbidity measurements (absorbance at 395 nm) were taken every minute to assess aggregation. A representative dataset is shown.

Next, we assessed foldamer specificity by testing whether they inhibited amyloidogenesis of the prion domain, NM, of yeast Sup35 [63]. (Arg-Benz)_4_-CONH_2_, (Arg-Sal)_3_-(Cit-Sal)-CONH_2_, Sal-(Lys-Sal)_3_-CONH_2_ and Ac-Sal-(Lys-Sal)_3_-CONH_2_ did not inhibit NM fibrillization (Figure 5A). In the presence of (Arg-Benz)_4_-CONH_2_, NM formed fibrils that exhibited greater ThT fluorescence (Figure 5A). EM revealed that purely NM fibrils formed in the presence or absence of (Arg-Benz)_4_-CONH_2_ and sedimentation analysis revealed that equal quantities of NM formed fibrils (results not shown). Thus, (Arg-Benz)_4_-CONH_2_ does not stimulate NM fibrillization. Rather, we suggest that NM accesses a different prion strain in the presence of (Arg-Benz)_4_-CONH_2_. NM accesses different prion strains in the presence of certain small molecules, such as EGCG [57,63]. None of these foldamers inhibited seeded NM fibrillization (results not shown). Thus, these foldamers are not generic inhibitors of amyloidogenesis.

### Sal-(Lys-Sal)_3_-CONH_2_ delays FUS aggregation

To further test specificity, we assessed inhibition of aggregation of FUS, an RNA-binding protein with a prion-like domain, which is connected with amyotrophic lateral sclerosis and frontotemporal dementia [1,58,64]. (Arg-Benz)_4_-CONH_2_, (Arg-Sal)_3_-(Cit-Sal)-CONH_2_ and Ac-Sal-(Lys-Sal)_3_-CONH_2_ did not inhibit FUS aggregation (Figure 5B). Interestingly, Sal-(Lys-Sal)_3_-CONH_2_ delayed FUS aggregation (Figure 5B). Sal-(Lys-Sal)_3_-CONH_2_ could serve as a lead foldamer to be optimized against FUS misfolding.

### (Arg-Sal)_3_-(Cit-Sal)-CONH_2_ and Ac-Sal-(Lys-Sal)_3_-CONH_2_ inhibit seeded Aβ42 fibrillization

**Figure 6:**
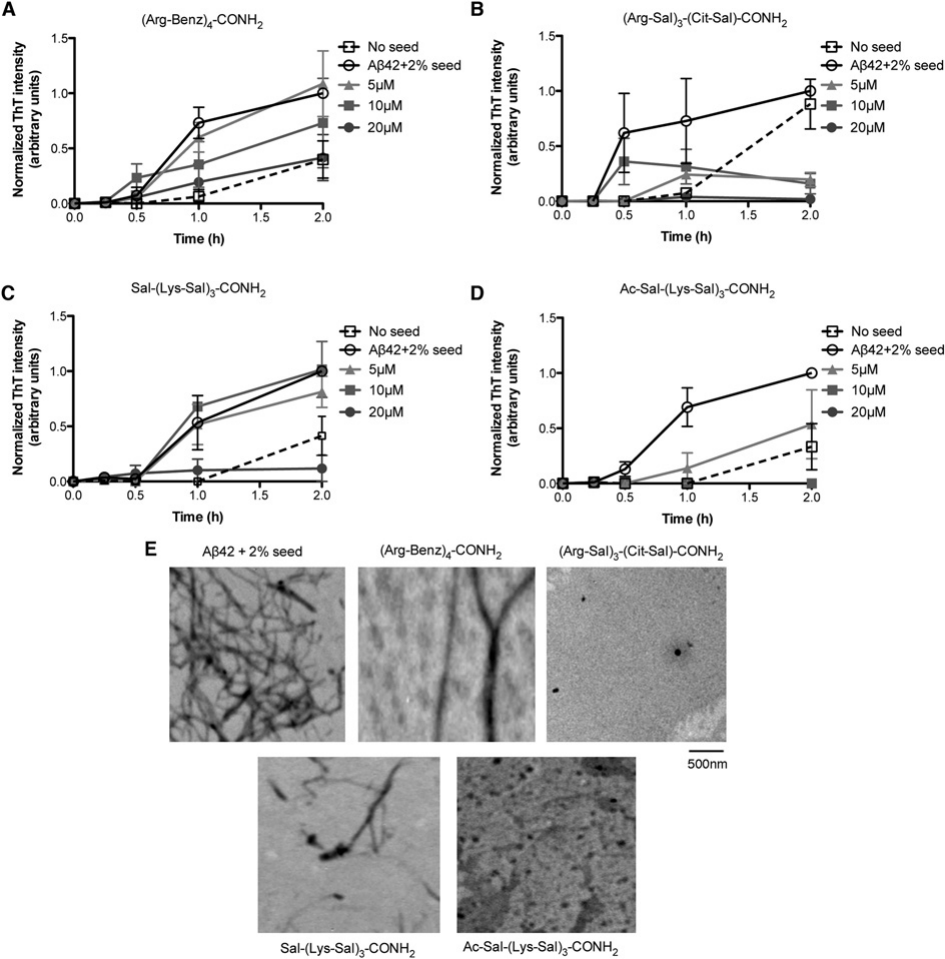
(Arg-Sal)_3_-(Cit-Sal)-CONH_2_ and Ac-Sal-(Lys-Sal)_3_-CONH_2_ inhibit seeded Aβ42 fibrillization (**A**–**D**) Aβ42 (5 μM) was incubated with agitation for 0–2 h at 25°C without (open squares) or with Aβ42 fibril seed (0.1 μM monomer) in the absence (open circles) or presence of 5 μM (filled triangles), 10 μM (filled squares) or 20 μM (filled circles) (Arg-Benz)_4_-CONH_2_ (**A**), (Arg-Sal)_3_-(Cit-Sal)-CONH_2_ (**B**), Sal-(Lys-Sal)_3_-CONH_2_ (**C**) or Ac-Sal-(Lys-Sal)_3_-CONH_2_ (**D**). Aβ42 fibrillization was assessed by ThT fluorescence. Values represent means±S.E.M. (*n*=3). (**E**) Aβ42 (5 μM) plus Aβ42 fibril seed (0.1 μM monomer) was incubated with agitation for 4 h at 25°C plus or minus the indicated foldamer (10 μM). Aβ42 fibrillization was assessed by EM. Scale bar, 500 nm.

(Arg-Benz)_4_-CONH_2_ and Sal-(Lys-Sal)_3_-CONH_2_ only inhibited seeded Aβ42 fibrillization when present at a 4-fold molar excess over Aβ42 (Figures 6A and 6C, filled circles; Figure 6E). Even at this high concentration, some fibrillization occurred in the presence of (Arg-Benz)_4_-CONH_2_ (Figure 6A, filled circles) but was very limited by Sal-(Lys-Sal)_3_-CONH_2_ (Figure 6C, filled circles). Thus, (Arg-Benz)_4_-CONH_2_ and Sal-(Lys-Sal)_3_-CONH_2_ are more potent inhibitors of spontaneous Aβ42 fibrillization (Figures 4A and 4C) than seeded Aβ42 fibrillization (Figures 6A and 6C). (Arg-Benz)_4_-CONH_2_ and Sal-(Lys-Sal)_3_-CONH_2_ likely preferentially inhibit the rearrangement of Aβ42 oligomers into fibril-nucleating species [22]. Once Aβ42 fibrils have formed, (Arg-Benz)_4_-CONH_2_ and Sal-(Lys-Sal)_3_-CONH_2_ have reduced ability to inhibit assembly.

Ac-Sal-(Lys-Sal)_3_-CONH_2_ and (Arg-Sal)_3_-(Cit-Sal)-CONH_2_ inhibited seeded Aβ42 fibrillization at all concentrations tested (Figures 6B, 6D and 6E). (Arg-Sal)_3_-(Cit-Sal)-CONH_2_ was more potent with an IC_50_ of ~2.5 μM (Figures 6B, 6D and 6E). Thus, Ac-Sal-(Lys-Sal)_3_-CONH_2_ and (Arg-Sal)_3_-(Cit-Sal)-CONH_2_ inhibit Aβ42 fibrillization even after formation of species that nucleate fibrillization.

### (Arg-Benz)_4_-CONH_2_ and (Arg-Sal)_3_-(Cit-Sal)-CONH_2_ inhibit spontaneous Aβ43 fibrillization

**Figure 7:**
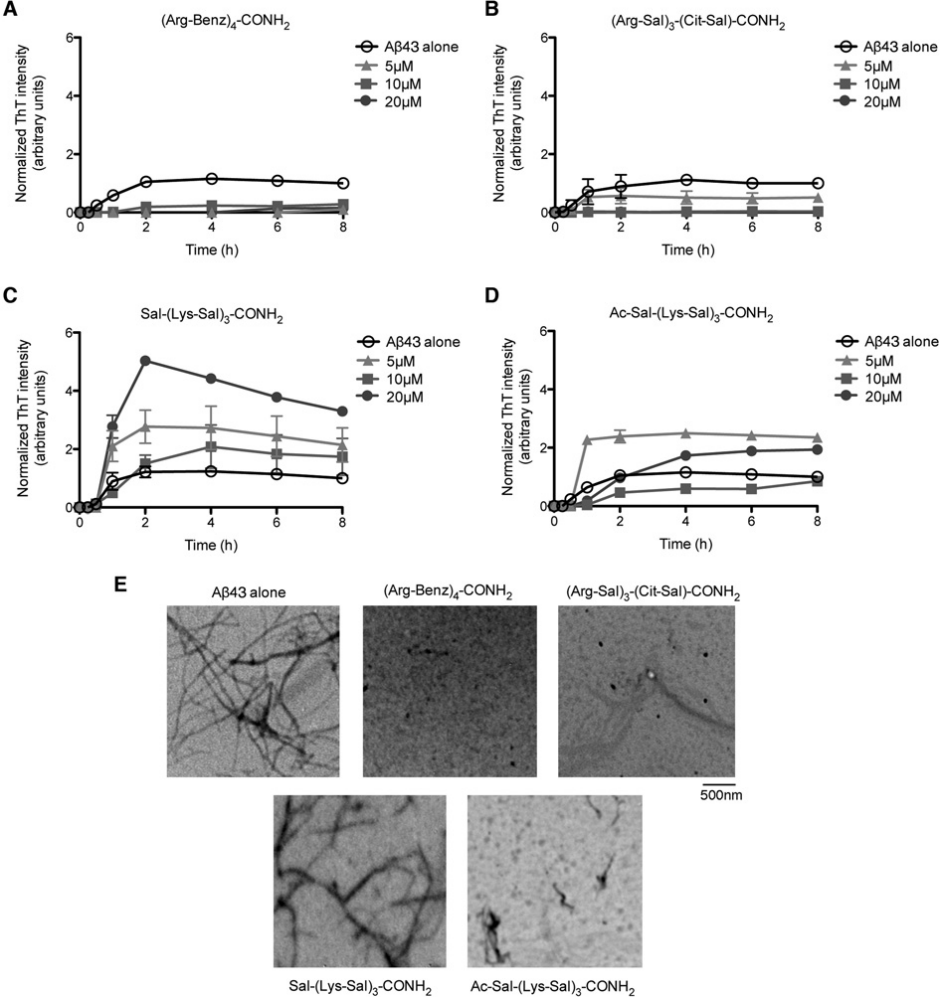
(Arg-Benz)_4_-CONH_2_ and (Arg-Sal)_3_-(Cit-Sal)-CONH_2_ inhibit spontaneous Aβ43 fibrillization (**A**–**D**) Aβ43 (5 μM) was incubated with agitation for 0–8 h at 25°C in the absence (open circles) or presence of 5 μM (filled triangles), 10 μM (filled squares) or 20 μM (filled circles) (Arg-Benz)_4_-CONH_2_ (**A**), (Arg-Sal)_3_-(Cit-Sal)-CONH_2_ (**B**), Sal-(Lys-Sal)_3_-CONH_2_ (**C**) or Ac-Sal-(Lys-Sal)_3_-CONH_2_ (**D**). Aβ43 fibrillization was assessed by ThT fluorescence. Values represent means±S.E.M. (*n*=3). (**E**) Aβ43 (5 μM) was incubated with agitation for 4 h at 25°C in the absence or presence of the indicated foldamer (10 μM). Aβ43 fibrillization was assessed by EM. Scale bar, 500 nm.

It is unknown whether inhibitors that target Aβ42 will also be active against Aβ43. In the absence of foldamer, Aβ43 fibrillization assembled more rapidly than Aβ42 (Figures 4A–4D, and 7A–7D). Sal-(Lys-Sal)_3_-CONH_2_ and Ac-Sal-(Lys-Sal)_3_-CONH_2_ did not block spontaneous Aβ43 fibrillization (Figures 7C–7E). Indeed, Sal-(Lys-Sal)_3_-CONH_2_ enabled Aβ43 fibrils to form that exhibited higher ThT fluorescence (Figures 7C and 7E) and sedimentation analysis revealed that equal quantities of Aβ43 formed fibrils (results not shown). Thus, Sal-(Lys-Sal)_3_-CONH_2_ does not stimulate Aβ43 fibrillization. Rather, Aβ43 may access a different amyloid strain in the presence of Sal-(Lys-Sal)_3_-CONH_2_. These findings suggest that potent inhibitors of spontaneous Aβ42 fibrillization may not inhibit spontaneous Aβ43 fibrillization. By contrast, (Arg-Benz)_4_-CONH_2_ and (Arg-Sal)_3_-(Cit-Sal)-CONH_2_ blocked spontaneous Aβ43 fibrillization (Figures 7A, 7B and E). In both cases, small oligomers were the major species (Figure 7E). The IC_50_ of (Arg-Sal)_3_-(Cit-Sal)-CONH_2_ was ~3.1 μM (Figures 7A and 7B).

### (Arg-Benz)_4_-CONH_2_ and (Arg-Sal)_3_-(Cit-Sal)-CONH_2_ inhibit seeded Aβ43 fibrillization

**Figure 8:**
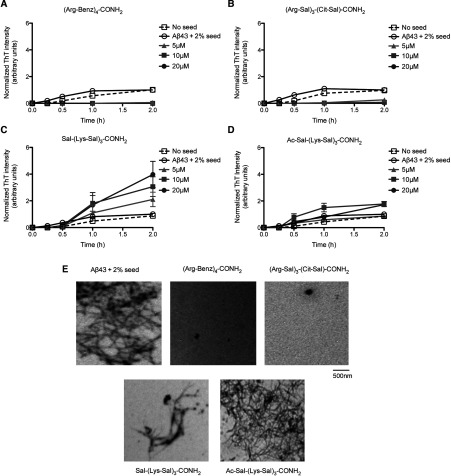
Foldamers (Arg-Benz)_4_-CONH_2_ and (Arg-Sal)_3_-(Cit-Sal)-CONH_2_ inhibit seeded Aβ43 fibrillization (**A**–**D**) Aβ43 (5 μM) was incubated with agitation for 0–2 h at 25°C without (open squares) or with Aβ43 fibril seed (0.1 μM monomer) in the absence (open circles) or presence of 5 μM (filled triangles), 10 μM (filled squares) or 20 μM (filled circles) (Arg-Benz)_4_-CONH_2_ (**A**), (Arg-Sal)_3_-(Cit-Sal)-CONH_2_ (**B**), Sal-(Lys-Sal)_3_-CONH_2_ (**C**) or Ac-Sal-(Lys-Sal)_3_-CONH_2_ (**D**). Aβ43 fibrillization was assessed by ThT fluorescence. Values represent means±S.E.M. (*n*=3). (**E**) Aβ43 (5 μM) plus Aβ43 fibril seed (0.1 μM monomer) was incubated with agitation for 4 h at 25°C plus or minus the indicated foldamer (10 μM). Aβ42 fibrillization was assessed by EM. Scale bar, 500 nm.

Aβ43 fibrils eliminated the lag phase of Aβ43 assembly (Figures 8A–8D, compare open squares and open circles). Sal-(Lys-Sal)_3_-CONH_2_ and Ac-Sal-(Lys-Sal)_3_-CONH_2_ did not inhibit seeded Aβ43 fibrillization (Figures 8C–8E). Sal-(Lys-Sal)_3_-CONH_2_ enabled Aβ43 to access fibrillar forms that generated a higher ThT fluorescence signal, perhaps indicative of a distinct Aβ43 amyloid strain (Figure 8C). By contrast, (Arg-Benz)_4_-CONH_2_ and (Arg-Sal)_3_-(Cit-Sal)-CONH_2_ blocked seeded Aβ43 fibrillization (Figures 8A, 8B and 8E). The IC_50_ of (Arg-Sal)_3_-(Cit-Sal)-CONH_2_ against seeded Aβ43 fibrillization was ~1.7 μM.

### Foldamers inhibit Aβ42 and Aβ43 fibrillization under different assembly conditions

**Figure 9:**
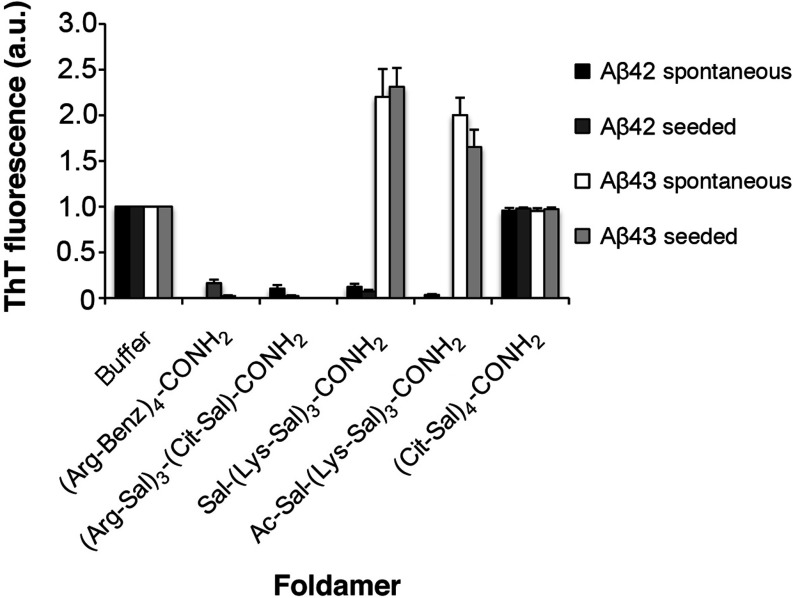
Foldamers inhibit Aβ42 and Aβ43 fibrillization under different assembly conditions Aβ42 or Aβ43 (5 μM) were incubated with agitation for 16 h at 25°C without or with Aβ42 fibril seed or Aβ43 fibril seed (0.1 μM monomer) plus or minus 20 μM (Arg-Benz)_4_-CONH_2_, (Arg-Sal)_3_-(Cit-Sal)-CONH_2_, Sal-(Lys-Sal)_3_-CONH_2_, Ac-Sal-(Lys-Sal)_3_-CONH_2_ or (Cit-Sal)_4_-CONH_2_. Fibrillization was assessed by ThT fluorescence. Values represent means±S.E.M. (*n*=3).

Next, we established that foldamers inhibited spontaneous and seeded Aβ42 and Aβ43 fibrillization under different assembly conditions, which might support formation of different amyloid strains. Thus, we avoided DMSO in Aβ preparation and assembled in a higher pH buffer. Under these conditions, a negative control foldamer, (Cit-Sal)_4_-CONH_2_, had no effect (Figure 9). By contrast, (Arg-Benz)_4_-CONH_2_, (Arg-Sal)_3_-(Cit-Sal)-CONH_2_, Sal-(Lys-Sal)_3_-CONH_2_ and Ac-Sal-(Lys-Sal)_3_-CONH_2_ inhibited spontaneous and seeded Aβ42 fibrillization (Figure 9), whereas only (Arg-Benz)_4_-CONH_2_ and (Arg-Sal)_3_-(Cit-Sal)-CONH_2_ inhibited spontaneous and seeded Aβ43 fibrillization (Figure 9).

### (Arg-Sal)_3_-(Cit-Sal)-CONH_2_ antagonizes formation of A11-reactive Aβ42 and Aβ43 oligomers

**Figure 10:**
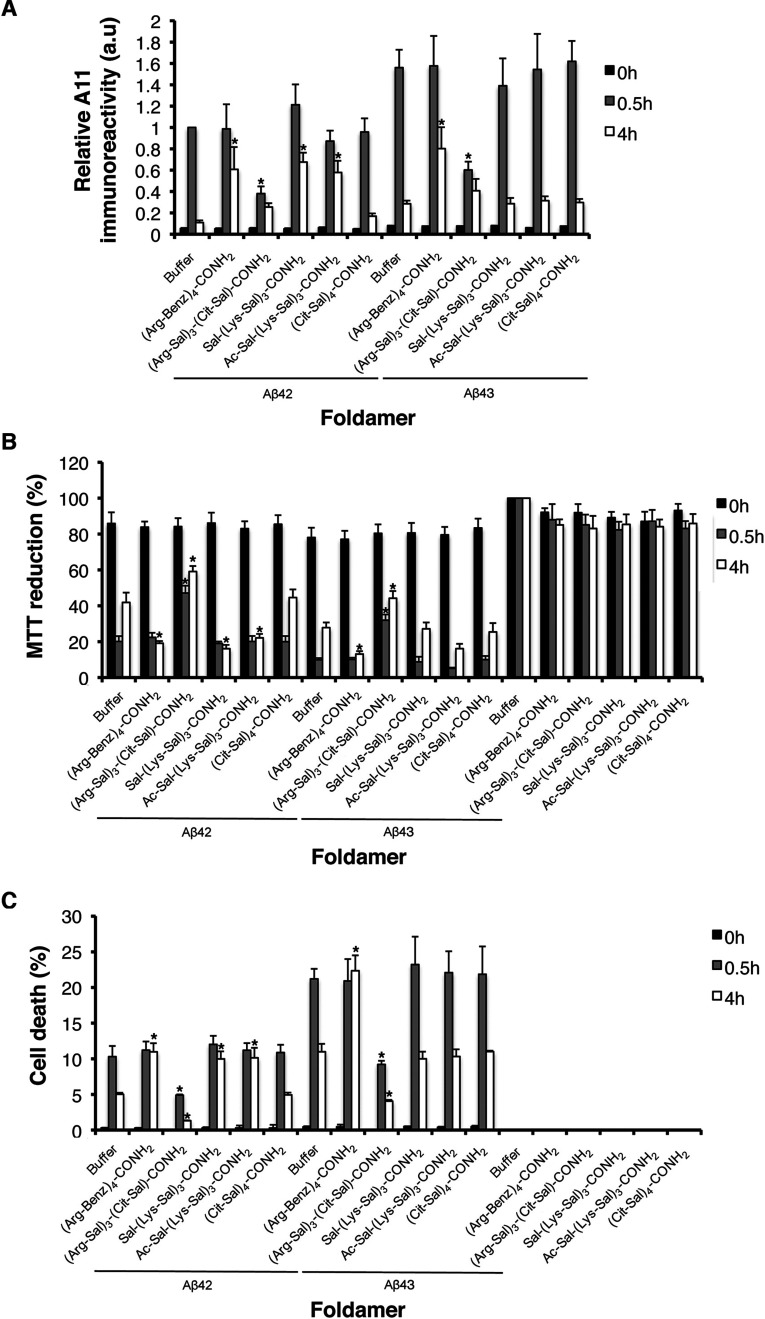
(Arg-Sal)_3_-(Cit-Sal)-CONH_2_ inhibits formation of toxic Aβ42 and Aβ43 conformers (**A**–**C**) Aβ42 or Aβ43 (5 μM) was incubated at 25°C with agitation for 0 h (black bars), 0.5 h (grey bars) or 4 h (white bars) in the absence or presence of 20 μM (Arg-Benz)_4_-CONH_2_, (Arg-Sal)_3_-(Cit-Sal)-CONH_2_, Sal-(Lys-Sal)_3_-CONH_2_, Ac-Sal-(Lys-Sal)_3_-CONH_2_ or (Cit-Sal)_4_-CONH_2_. At the indicated times, the amount of A11-reactive species present (**A**) or toxicity to SH-SY5Y neuroblastoma cells in culture was determined via MTT reduction (**B**) or LDH release (**C**). We also assessed the toxicity of buffer, (Arg-Benz)_4_-CONH_2_, (Arg-Sal)_3_-(Cit-Sal)-CONH_2_, Sal-(Lys-Sal)_3_-CONH_2_, Ac-Sal-(Lys-Sal)_3_-CONH_2_ or (Cit-Sal)_4_-CONH_2_ alone (**B** and **C**). Values represent means±S.E.M. (*n*=3). A one-way ANOVA with the post-hoc Dunnett's multiple comparisons test was used to compare Aβ42 plus buffer to each Aβ42 plus foldamer condition (* denotes *P*< 0.05). Likewise, a one-way ANOVA with the post-hoc Dunnett's multiple comparisons test was used to compare Aβ43 plus buffer to each Aβ43 plus foldamer condition (* denotes *P*< 0.05).

Could foldamers inhibit the formation of toxic Aβ42 and Aβ43 oligomers? To assess toxic Aβ42 and Aβ43 oligomer formation, we employed the conformation-specific A11 antibody, which specifically recognizes preamyloid oligomers formed by multiple proteins, including Aβ42, but not monomers or fibrils [21]. We assessed formation of A11-reactive species at the start of spontaneous assembly (0 h), at the end of lag phase (0.5 h), and at the endpoint of fibrillization (4 h). In the absence of Aβ42 and Aβ43, no A11 immunoreactivity was observed (results not shown). For Aβ42 and Aβ43, A11-reactive conformers were scarce at the start of the reaction (Figure 10A, buffer controls, black bars), abundant at end of lag phase (Figure 10A, buffer controls, grey bars), and declined once fibrillization was complete (Figure 10A, buffer controls, white bars). Aβ43 exhibited greater A11-immunoreactivity than Aβ42 and appears more prone to accessing this toxic conformation (Figure 10A).

A negative control foldamer, (Cit-Sal)_4_-CONH_2_ (Figure 2), had no effect on the appearance and disappearance of A11-reactive Aβ42 and Aβ43 conformers (Figure 10A). (Arg-Benz)_4_-CONH_2_, Sal-(Lys-Sal)_3_-CONH_2_ and Ac-Sal-(Lys-Sal)_3_-CONH_2_ had no effect on the abundance of A11-reactive Aβ42 or Aβ43 oligomers after 0.5 h (Figure 10A, grey bars). Thus, these foldamers inhibit spontaneous Aβ42 or Aβ43 fibrillization without affecting the formation of A11-reactive conformers. Furthermore, after 4 h in the presence of (Arg-Benz)_4_-CONH_2_, Sal-(Lys-Sal)_3_-CONH_2_ and Ac-Sal-(Lys-Sal)_3_-CONH_2_, A11-reactive Aβ42 species remained at higher levels and did not decline as much as they did in the absence of foldamer (Figure 10A, white bars). Thus, (Arg-Benz)_4_-CONH_2_, Sal-(Lys-Sal)_3_-CONH_2_, and Ac-Sal-(Lys-Sal)_3_-CONH_2_ stabilize A11-reactive conformers. (Arg-Benz)_4_-CONH_2_, but not Sal-(Lys-Sal)_3_-CONH_2_ or Ac-Sal-(Lys-Sal)_3_-CONH_2_, had a similar effect on A11-reactive Aβ43 species (Figure 10A). By contrast, A11-reactive Aβ43 species declined more extensively after 4 h in the presence of Sal-(Lys-Sal)_3_-CONH_2_ or Ac-Sal-(Lys-Sal)_3_-CONH_2_ (Figure 10A, white bars), which do not inhibit spontaneous Aβ43 fibrillization (Figures 7C and 7D).

(Arg-Sal)_3_-(Cit-Sal)-CONH_2_ inhibited the formation of A11-reactive Aβ42 and Aβ43 conformers after 0.5 h (Figure 10A, grey bars). After 4 h, (Arg-Sal)_3_-(Cit-Sal)-CONH_2_ prevented further accumulation of A11-reactive Aβ42 and Aβ43 conformers (Figure 10A, white bars). Thus, (Arg-Sal)_3_-(Cit-Sal)-CONH_2_ inhibits fibrillization as well as toxic oligomer formation by Aβ42 and Aβ43. (Arg-Sal)_3_-(Cit-Sal)-CONH_2_ might inhibit Aβ42 and Aβ43 misfolding by a mechanism that is distinct to the other foldamers and arrests Aβ42 and Aβ43 misfolding prior to an A11-reactive oligomeric state.

### (Arg-Sal)_3_-(Cit-Sal)-CONH_2_ inhibits formation of toxic Aβ42 and Aβ43 conformers

Next, we evaluated the relative toxicity of Aβ42 and Aβ43 conformers formed in the absence or presence of foldamers. We applied Aβ42 and Aβ43 conformers to SH-SY5Y neuroblastoma cells and assessed cell viability using MTT reduction and LDH release. Foldamers and buffer display little toxicity in the absence of Aβ (Figures 10B and 10C, far right). In the absence of foldamer, Aβ42 and Aβ43 exhibited little toxicity after 0 h (Figures 10B and 10C), consistent with reduced A11 immunoreactivity at this time (Figure 10A). Aβ42 and Aβ43 were more toxic after 0.5 h of assembly than after 4 h (Figures 10B and 10C), indicating that conformers that accumulate at the end of lag phase are more toxic than mature fibrils. In the absence of foldamer, Aβ43 conformers were generally more toxic than Aβ42 conformers (Figures 10B and 10C). The negative control foldamer, (Cit-Sal)_4_-CONH_2_, had no effect on toxicity (Figures 10B and 10C). (Arg-Benz)_4_-CONH_2_, Sal-(Lys-Sal)_3_-CONH_2_ and Ac-Sal-(Lys-Sal)_3_-CONH_2_ had no effect on the toxicity of Aβ42 conformers after 0.5 h of assembly (Figures 10B and 10C, grey bars), but after 4 h of assembly the toxicity of Aβ42 conformers was enhanced (Figures 10B and 10C, white bars). Thus, (Arg-Benz)_4_-CONH_2_, Sal-(Lys-Sal)_3_-CONH_2_ and Ac-Sal-(Lys-Sal)_3_-CONH_2_ inhibit spontaneous Aβ42 fibrillization such that more toxic conformers are maintained (Figures 10A–C). For Aβ43, neither Sal-(Lys-Sal)_3_-CONH_2_ nor Ac-Sal-(Lys-Sal)_3_-CONH_2_ affected the toxicity of conformers after 0.5 h or 4 h (Figures 10B and 10C). However, as for Aβ42, (Arg-Benz)_4_-CONH_2_ had no effect on the toxicity of Aβ43 conformers after 0.5 h of assembly (Figures 10B and 10C, grey bars), but after 4 h the toxicity of Aβ43 conformers was enhanced (Figures 10B and 10C, white bars). Thus, (Arg-Benz)_4_-CONH_2_ inhibits spontaneous Aβ43 fibrillization in a manner that maintains toxic conformers (Figures 10A–10C).

(Arg-Sal)_3_-(Cit-Sal)-CONH_2_, which inhibited the formation of A11-reactive Aβ42 and Aβ43 conformers after 0.5 h (Figure 10A, grey bars), also partially reduced the toxicity of Aβ42 and Aβ43 conformers at this time (Figures 10B and 10C, grey bars) and at 4 h (Figures 10B and 10C, white bars). Although Aβ42 and Aβ43 conformers still conferred toxicity in comparison with buffer controls, (Arg-Sal)_3_-(Cit-Sal)-CONH_2_ was the only foldamer that antagonized Aβ42 and Aβ43 toxicity.

(Arg-Sal)_3_-(Cit-Sal)-CONH_2_ inhibits spontaneous and seeded Aβ42 and Aβ43 fibrillization and reduces accumulation of toxic Aβ42 and Aβ43 conformers. This combination of properties could have therapeutic potential for three reasons. First, (Arg-Sal)_3_-(Cit-Sal)-CONH_2_ antagonizes Aβ42 as well as Aβ43, which is an often overlooked but highly toxic Aβ species [13–16]. Secondly, (Arg-Sal)_3_-(Cit-Sal)-CONH_2_ inhibits the formation of toxic Aβ42 and Aβ43 conformers, which could reduce localized neurodegeneration [65]. Thirdly, (Arg-Sal)_3_-(Cit-Sal)-CONH_2_ inhibits seeded Aβ42 and Aβ43 assembly, which could prevent the spreading of Aβ pathology throughout the brain in AD [29–31]. Further studies are needed to assess the utility of (Arg-Sal)_3_-(Cit-Sal)-CONH_2_ against Aβ misfolding and toxicity in the metazoan nervous system.

Future studies will reveal the mechanisms by which foldamers antagonize Aβ-misfolding. Foldamers have amides oriented appropriately (Figure 2) to block growth from fibril ends during seeded polymerization. They are also relatively flat and aromatic (Figure 2) and might antagonize secondary nucleation by binding to the lateral surface of fibrils. Foldamer insertion into molten oligomers could inhibit rearrangement events required for nucleation during spontaneous assembly. Differences in the ability of specific foldamers to inhibit Aβ42 fibrillization compared with Aβ43 fibrillization probably reflect differential antagonism of events driven by the additional C-terminal steric zipper hexapeptide (G^38^VVIAT^43^) of Aβ43.

Aromatic foldamers could be useful amyloidogenesis inhibitors for various disease-associated proteins. Indeed, another class of aromatic foldamer inhibits amylin fibrillization, which is connected to Type 2 diabetes [66]. Thus, foldamers await further development to antagonize protein misfolding in several settings.
